# Platelet to Lymphocyte Ratio and Neutrophil to Lymphocyte Ratio May Contribute Little Compared to Standard Preoperative Evaluation

**DOI:** 10.21470/1678-9741-2018-0242

**Published:** 2018

**Authors:** Renato Abdala Karam Kalil, Felipe Borsu de Salles

**Affiliations:** 1 Instituto de Cardiologia do Rio Grande do Sul, Fundação Universitária de Cardiologia, Porto Alegre, RS, Brazil.; 2 Instituto de Cardiologia do Rio Grande do Sul, Porto Alegre, RS, Brazil.

Dear Editor,

A recent paper published in Brazilian Journal of Cardiovascular Surgery (BJCVS) introduces platelet to lymphocyte ratio (PLR) and neutrophil to lymphocyte ratio (NLR) as two novel index to predict development of postoperative acute kidney injury (AKI) of isolated coronary artery bypass grafting^[1]^. This case-control study included a wide range of patients with AKI (*i.e*. increase of 0.3 mg/dl in serum creatinine) despite most paper having considered only dialysis as AKI. Higher risks patients - critical patients, renal impairment, left ventricular systolic dysfunction, etc - where excluded from this study, even though they are more susceptible to develop AKI and to benefit more from the development of better discriminating tools.

There is no mention of sample size calculation, which may hinder a proper analysis of data. The difference of diabetes (46% *vs*. 34%) and smoking history (46% *vs*. 37%) prevalence between groups may not have come out statistically different due to the small size of the sample. In addition, the statistical difference of renal function (serum creatinine and urea) and inflammatory (C-reactive protein) biomarkers between groups shows that AKI group already had worse renal function despites what these novel indexes demonstrate. Moreover, multivariate analysis showed that creatinine had higher odds ratio than PLN and NLR, *id est* these new indexes were inferior to traditional and widely used creatinine, and may contribute little compared to standard preoperative evaluation.
